# Fall Prevention in Community Settings: Results from Implementing *Stepping On* in Three States

**DOI:** 10.3389/fpubh.2014.00232

**Published:** 2015-04-27

**Authors:** Marcia G. Ory, Matthew Lee Smith, Luohua Jiang, Robin Lee, Shuai Chen, Ashley D. Wilson, Judy A. Stevens, Erin M. Parker

**Affiliations:** ^1^Department of Health Promotion and Community Health Sciences, Texas A&M Health Science Center, College Station, TX, USA; ^2^Department of Health Promotion and Behavior, The University of Georgia College of Public Health, Athens, GA, USA; ^3^Department of Epidemiology, University of California, Irvine, CA, USA; ^4^National Center for Injury Prevention and Control, Centers for Disease Control and Prevention, Atlanta, GA, USA; ^5^Department of Statistics, Texas A&M University, College Station, TX, USA

**Keywords:** fall prevention, evidence-based program, *Stepping On*, older adult

## Abstract

*Stepping On* is a community-based intervention that has been shown in a randomized controlled trial to reduce fall risk. The Wisconsin Institute for Healthy Aging adapted *Stepping On* for use in the United States and developed a training infrastructure to enable dissemination. The purpose of this study is to: (1) describe the personal characteristics of *Stepping On* participants; (2) quantify participants’ functional and self-reported health status at enrollment, and (3) measure changes in participants’ functional and self-reported health status after completing the program. Both survey and observed functional status [timed up and go (TUG) test] data were collected between September 2011 and December 2013 for 366 participants enrolled in 32 *Stepping On* programs delivered in Colorado, New York, and Oregon. Paired *t*-tests and general estimating equations models adjusted for socio-demographic factors were performed to assess changes over the program period. Among the 266 participants with pre–post survey data, the average participant age was 78.7 (SD ± 8.0) years. Most participants were female (83.4%), white (96.9%), and in good health (49.4%). The TUG test scores decreased significantly (*p* < 0.001) for all 254 participants with pre–post data. The change was most noticeable among high risk participants where TUG time decreased from 17.6 to 14.4 s. The adjusted odds ratio of feeling confident about keeping from falling was more than three times greater after completing *Stepping On*. Further, the adjusted odds ratios of reporting “no difficulty” for getting out of a straight back chair increased by 89%. Intended for older adults who have fallen in the past or are afraid of falling, *Stepping On* has the potential to reduce the frequency and burden of older adult falls.

## Introduction

Although older adults fall more frequently than younger people, falls are not a normal part of aging (1). Over the past three decades, researchers have identified the major modifiable fall risk factors as well as effective fall interventions (2–4). Some interventions shown to be effective in randomized control trials have been translated into programs and implemented in community settings. One such program is *Stepping On*, which was developed in Australia (5) and later adapted for use in the United States by the Wisconsin Institute for Healthy Aging (WIHA). The WIHA now provides training for *Stepping On* leaders as well as an implementation manual and evaluation plan (6, 7).

*Stepping On* is a group program proven to reduce falls and build confidence in ambulatory older adults who have fallen previously or are afraid of falling (8). A randomized trial of *Stepping On* found that participants’ risk of falling was approximately 30% lower than those who did not receive the intervention (5). Stevens (2014) noted that a recent analysis also found that *Stepping On* showed a positive return on investment of 59% (J. Stevens, CDC, personal communication. 8/1/2014).

As described in the WIHA Implementation Manual (6), the program is delivered by a trained leader and a peer leader, who apply adult education and social learning principles to teach older adults about fall risk factors and strategies to reduce their fall risk. The traditional program consists of a group of 10–14 participants attending a 2 hour session held once a week for seven consecutive weeks. Content is provided by the program leaders and by invited health professional “guest experts.” During the program, older adults learn how to improve their balance and strength, increase their safety at home and in the community, and the importance of vision assessment and medication reviews.

In 2011, the Centers for Disease Control and Prevention (CDC) launched a 5-year project funding State Departments of Health to implement *Stepping On* in selected communities in Oregon, Colorado, and New York. This was part of a larger project in which the CDC funded these states to reduce falls and fall-related injuries by engaging fall prevention coalitions, healthcare organizations, and other partners to implement evidence-based fall prevention programs in clinical and community settings. *Stepping On* is intended for older adults with moderate fall risk, such as an older adult who fell in the past year or is afraid of falling. Additional information about CDC’s fall prevention initiative can be found elsewhere (9).

This manuscript describes the results of implementing *Stepping On* during the first 2 years of the project. The purposes were to: (1) describe the personal characteristics and session attendance of *Stepping On* participants; (2) quantify participants’ functional and self-reported health status at enrollment, and (3) measure changes in participants’ functional and self-reported health status after completing the program.

## Materials and Methods

### Program planning and participant recruitment

The WIHA offered training for master trainers who, in turn, trained local group facilitators. State program leads (i.e., designated contacts at the State Departments of Health) recommended facilitators who were part of local public health or aging services delivery systems. Following the *Implementation Guide* (6), state program leads and facilitators worked together to identify appropriate sites for *Stepping On* programs.

Program participants were recruited through a variety of channels, including distributing flyers, conducting informational presentations, making personal contact in places where older adults congregated such as senior centers, recreation centers, or senior housing or retirement homes, as well as through contacts with their health care providers and television, newspaper, and radio advertisements.

*Stepping On* staff used a standardized admission form and screening questions to identify appropriate participants. To be eligible, a participant needed to be 60 years of age or older, live independently in the community, and be able to walk without the help of another person or with an assistive device (e.g., walker, scooter). Although some information about chronic illnesses was obtained during the screening process, information on the number and type of chronic conditions was not systematically collected as part of the evaluation survey.

In preparation for program delivery, each state conducted training sessions for *Stepping On* program leaders. The program was delivered in multiple settings, including healthcare organizations, senior housing or assisted living facilities, faith-based organizations, recreational facilities, and senior centers. Additional information about program preparation, implementation, and evaluation can be found in the *Stepping On* *Implementation Guide* (6).

### Data collection

Data were collected from multiple sources. Attendance was recorded at each session and these records were used to describe participant retention over the 7 week program. Program completion was defined as attending five of the seven sessions. A 20-question self-administered questionnaire was used to collect data at the initial *Stepping On* session (enrollment or baseline survey) and at the last session (program completion or post-intervention survey). Each survey took about 15 minutes to complete and assistance was provided to participants who needed help filling out the forms. Survey questions included participant characteristics (e.g., age group, gender, race, ethnicity), general health status (excellent, very good, good, fair, and poor), and whether the participant had been referred to the program by a healthcare provider. Also measured were satisfaction with their current physical activity level (very, mostly, somewhat, or not at all satisfied) and confidence in their ability to keep themselves from falling (five-point scale ranging from strongly agree to strongly disagree). Self-reported functional ability was assessed by the reported level of difficulty in performing various activities (e.g., climbing one flight of stairs) on a four-point scale ranging from (1) no difficulty to (4) unable to do (10).

The *Timed Up and Go* (TUG) test was used to measure functional status at the first and last *Stepping On* sessions. This test has been widely used to assess functional mobility and predict fall risk (11, 12) and has been validated among community-dwelling older adults (13). The test measures the time in seconds for a participant to “stand up from a standard arm chair, walk at [his or her] typical or normal pace to a line on the floor 3 m away, turn, return, and sit down again” (14). Participants who completed the TUG in <12 s were classified as having low fall risk and those who took 12 or more seconds were classified as high risk (15).

The Texas A&M University Institutional Review Board granted approval to analyze secondary data on program participants and outcomes collected using survey instruments and functional assessments.

### Statistical analyses

To identify potential biases from loss to follow-up, we used the chi-square test to compare participant characteristics, number of sessions attended, and TUG results from participants who completed both the baseline and post-intervention surveys to those who only completed the baseline survey (were lost to follow-up). Two-tailed paired *t*-tests were used to compare participant’s TUG results at the start and end of the program. General estimating equation (GEE) models using a logit link function were used to compare self-reported health status, satisfaction with activity levels, confidence in not falling, and self-reported functional status indicators at the start and end of the program. GEE models are longitudinal data models that use all available data in model estimation (i.e., do not require paired data) and can account for the correlation among repeated measures from the same participant. Each GEE model controlled for age group, gender, race, and program location. All models were run using SAS version 9.3 (SAS Institute Inc., Cary, NC, USA).

## Results

### Program implementation and participant characteristics

Between September 1, 2011 and December 31, 2013, the three states hosted four *Stepping On* training sessions. There were 64 leaders trained and 32, 7-week *Stepping On* programs delivered. Four hundred nineteen participants aged 60 years and older enrolled and 336 participants (80.2%) completed the enrollment or baseline survey. Of these, 274 (81.5%) participants attended five or more sessions and 138 (41.1%) attended all seven sessions.

As indicated in Table 1, of the 336 participants who completed the baseline survey, 60 (17.9%) attended programs in Oregon, 91 (27.1%) in Colorado, and 185 (55.1%) in New York (Table 1). The age distribution was similar among participants in each state. The mean age was 78.7 (SD ± 8.0) years. Overall, the majority of people who enrolled were female (83.3%), white (96.0%), and non-Hispanic (97.9%). The majority of participants reported good (50.3%) or excellent to very good health (34.1%). Only 22 (6.7%) participants were referred to *Stepping On* by a healthcare provider.

**Table 1:** **Characteristics of *Stepping On* participants**.

	All enrolled participants^a^	Participants who completed both the baseline enrollment and post-intervention surveys	Participants who completed only the baseline enrollment survey		
			
	*N* = 336	*n* = 266	*n* = 70	
			
	*N* (%)	*N* (%)	*N* (%)	*X*^2^	*p*
**Program location**				0.90	0.639
Oregon	60 (17.9)	45 (16.9)	15 (21.4)
Colorado	91 (27.1)	74 (27.8)	17 (24.3)		
New York	185 (55.1)	147 (55.3)	38 (54.3)		
**Age group**				0.58	0.749
60–69	53 (15.8)	44 (16.5)	9 (12.9)
70–79	119 (35.4)	93 (35.0)	26 (37.1)		
80+	164 (48.8)	129 (48.5)	35 (50.0)		
**Gender**				0.01	0.914
Female	279 (83.3)	221 (83.4)	58 (82.9)
Male	56 (16.7)	44 (16.6)	12 (17.1)		
Missing	1	1	0		
**Race**				2.61	0.106
White	316 (96.0)	253 (96.9)	63 (92.7)
Non-white	13 (4.0)	8 (3.1)	5 (7.4)		
Missing	7	5	2		
**Ethnicity (Hispanic/Latino)**				2.07	0.151
Hispanic	7 (2.1)	4 (1.5)	3 (4.4)
Non-Hispanic	322 (97.9)	256 (98.5)	66 (95.7)		
Missing	7	6	1		
**General health status**				3.09	0.214
Excellent or Very Good	114 (34.1)	96 (36.2)	18 (26.1)
Good	168 (50.3)	131 (49.4)	37 (53.6)	
Fair/Poor	52 (15.6)	38 (14.3)	14 (20.3)	
Missing	2	1	1	
**Referred to program by healthcare provider**	22 (6.7)	19 (7.2)	3 (4.6)	0.57	0.452
Missing	8	3	5	
**Timed up and go (tug) time at enrollment**				0.78	0.378
Low risk (enrollment TUG <12 s)	165 (50.3%)	135 (51.5%)	30 (45.5%)
High risk (enrollment TUG ≥12 s)	163 (49.7%)	127 (48.5%)	36 (54.6%)	
Missing	8	4	4	
**Participants who completed 70%+ of sessions**	274 (81.6%)	252 (94.7%)	22 (31.4%)	147.60	<0.001

*^a^Enrolled participants include all persons 60 years and older who filled out the baseline survey on the first day of the program (336/419 *Stepping On* participants). Individual survey questions may have had missing data*.

There were 266 (63.5%) participants who completed both the baseline and post-intervention surveys; 70 completed only the baseline survey and were considered drop outs. Among the 266 participants with pre-post survey data, the average participant age was 78.7 (SD ± 8.0) years. Most participants were female (83.4%), white (96.9%), and in at least good health (85.6%). The majority of the participants with baseline and post-intervention surveys (94.7%) completed 70% of the seven session program. There were no statistically significant differences between those who completed both surveys (the analytical sample) and those who only completed the baseline survey except in terms of class completion (Table 1).

### Participant functional performance

Of 336 participants with baseline data, 254 (75.6%) completed the TUG test at both baseline and post-intervention (Table 2). Of these, 123 (48.4%) were classified as high risk. After completing *Stepping On*, overall TUG scores significantly decreased 2.1 s (SD ± 3.1). The change was greatest among high risk participants whose TUG scores decreased an average of 3.2 s (SD ± 3.9).

**Table 2:** **Changes in *Stepping On* participants’ timed up and go (TUG) times in seconds from baseline to post-intervention**^a^.

	Baseline TUG		Post-intervention TUG		Change in TUG from baseline to post-intervention^b^		
Changes in timed up and go (TUG) times (in seconds)	*N*	Mean (±SD)	*N*	Mean (±SD)	*N*	Mean (±SD)	*p*-value
TUG times for all participants	254	13.5 (±5.7)	254	11.4 (±4.7)	254	−2.1 (±3.1)	<0.001
High risk (enrollment TUG time ≥12 s)	123	17.6 (±5.6)	123	14.4 (±4.9)	123	−3.2 (±3.9)	<0.001
Low risk (enrollment TUG time < 12 s)	131	9.6 (±1.4)	131	8.6 (±1.8)	131	−1.0 (±1.5)	<0.001

*SD, standard deviation*.

*^a^While 329 participants completed the TUG at enrollment, this table highlights the 254 participants who completed the TUG at both baseline and post-intervention*.

*^b^Paired *t*-tests with an alpha of 0.05 were used to compare changes in participant’s TUG time between baseline and post-intervention. A reduction in time indicates a positive functional improvement*.

### Self-reported health and functional outcomes

Table 3 compares self-reported health and functional outcomes at baseline enrollment and post-intervention. Odds ratios were adjusted for gender, age, race, and state. The adjusted odds ratio (aOR) of reporting excellent or very good health status increased by 56% (aOR = 1.56, 95% CI 1.22–2.00). The odds of being very or mostly satisfied with their physical activity levels increased significantly (aOR = 1.74, 95% CI 1.36–2.23) as did their confidence that a fall could be avoided (aOR = 4.60, 95% CI 2.94–7.22). Three of the five items assessing functional status indicated improvement (Table 3). Participants were more likely to report no difficulty in walking one block (aOR = 1.36, 95% CI 1.09, 1.69); getting out of a straight backed chair (aOR = 1.89, 95% CI 1.43–2.50); and climbing one flight of stairs (aOR = 1.42, 95% CI 1.11–1.82). Controlling for the number of sessions attended did not substantially affect our results (data not shown).

**Table 3:** **Changes in *Stepping On* participants’ self-reported health and functional outcomes from baseline to post-intervention**^a^.

Self-reported health and functional outcome measures	Baseline (*N* = 266)^b^	Post-intervention (*N* = 266)^b^	Adjusted change from baseline to post-intervention^c^	
	*N* (%)	*N* (%)	Odds ratios from logistic models	*p*-value
**Health status, satisfaction, and confidence**
Excellent or very good health status	96 (36.2%)	123 (46.8%)	1.56 (1.22, 2.00)	<0.001
Very/mostly satisfied with physical activity levels	123 (46.8%)	155 (59.4%)	1.74 (1.36, 2.23)	<0.001
Feel confident not falling (strongly agree or agree)	180 (69.8%)	237 (91.2%)	4.60 (2.94, 7.22)	<0.001
**Self-reported functional status**
No difficulty in walking across room	195 (75.0%)	204 (79.4%)	1.23 (0.95, 1.59)	0.121
No difficulty in walking one block	144 (55.8%)	161 (62.4%)	1.36 (1.09, 1.69)	0.007
No difficulty in stooping, crouching, kneeling	59 (23.0%)	66(25.8%)	1.12 (0.86, 1.46)	0.403
No difficulty in getting out of a straight back chair	154 (59.7%)	189 (73.3%)	1.89 (1.43, 2.50)	<0.001
No difficulty in climbing one flight of stairs	102 (40.2%)	125 (48.6%)	1.42 (1.11, 1.82)	0.006

*^a^Data are reported for the *n* = 266 participants who completed both the baseline and post-intervention surveys*.

*^b^The sample size is slightly smaller than 266 for some health outcomes due to missing data on individual outcome measures. The amount of missing data ranges from 0 to 5% for different outcomes*.

*^c^Adjusted odds ratios from GEE logistic regression modeling the probability of response = 1 at an alpha of 0.05. All models account for repeated measures from the same participant and are adjusted for gender, age, race, and program location. An odds ratio >1 represents a positive improvement in self-reported health*.

## Discussion

This study examined 2 years of evaluation data collected from older adults aged 60 years and older who participated in the *Stepping On* community-based fall prevention program. We observed improvements in both the observed and self-reported functional abilities of program participants. Comparing data collected at baseline enrollment and program completion, *Stepping On* was associated with significant improvements in TUG scores and in self-reported measures of health status, satisfaction with their physical activity levels, and fall-related confidence. This suggests that *Stepping On* contributes to functional improvements and may also contribute to participants’ general sense of well-being. The largest improvement was seen in feeling confident that falls could be avoided, which increased from approximately 70% at enrollment to over 90% after completion of the *Stepping On* program. Given that fear of falling is a fall risk factor (16–19), reduced fear coupled with increased functional ability is important components of an effective fall prevention program.

Recruitment and retention of participants is a concern for most fall prevention programs. While the race and ethnicity of *Stepping On* participants reflected the population from which they were recruited, there was a low percentage of male participants. There were limited numbers of referrals from health care providers, which suggest the need for better linkages between clinical and community approaches to fall prevention (20). Involvement of health care professionals can be critical for motivating older patients at risk of falling to enroll in and complete evidence-based fall prevention programs.

In regards to participant retention, we observed some attrition; however, the majority of the 366 enrolled participants (81.5%) completed at least 70% of the sessions. *Stepping On* runs only 7 weeks, so program attrition may be less of a problem than for longer running programs. For example, the fall prevention program, Tai Chi Moving for Better Balance (TCMBB), requires two 1 hour sessions over the course of 12 weeks (21). For TCMBB, only about half of the participants completed at least 70% of the program sessions (22). It also may have helped that *Stepping On* includes a social component, a break halfway through the 2 hour session, when participants can mingle and share refreshments. Further, it is possible that using the TUG test may have helped retain participants. While no data were systematically collected on participants like or dislike of the TUG test, multiple participants told their leaders that they enjoyed receiving their TUG times. The importance of timely performance feedback has been documented previously as a motivating factor for program participation (23).

### Limitations

This study has a number of limitations that must be acknowledged. Participants were self-selected and this may limit the generalizability of the results to the broader older adult population in those communities. As we did not collect data on co-morbid conditions, we could not determine if our participants were similar to the broader population of older adults who were fearful of falling or had experienced prior falls. Similarly, we were unable to assess the extent to which co-morbid conditions were related to our study outcomes.

In order to minimize the reporting burden on the program implementation staff, we used a limited number of self-reported outcomes and one timed functional assessment (i.e., the TUG test). Although there was training provided for conducting the TUG (24), including available step-by-step online videos, this training was limited. Therefore, results may not be comparable to standardized TUG tests administered by trained professionals and some misclassification of a participant’s fall risk may have occurred. While participants reported improvements in self-reported functional ability and demonstrated better TUG scores, we do not know if there was a reduction in falls. Data about falls were not collected because of anticipated problems with recall bias.

Although we did not assess fidelity directly, we believe that program fidelity was maintained by training and certifying group facilitators and using the detailed *Implementation Guide* that emphasized the importance of program fidelity.

## Conclusion

*Stepping On* was previously shown to be effective at reducing fall risk in a randomized controlled trial. Intended for older adults who have fallen in the past or are afraid of falling, *Stepping On* applies adult education and social learning principles to teach older adults strategies that they can use to reduce their risk of falling. *Stepping On* participants practice balance and strength exercises, learn how to increase their safety at home and in the community, and learn about the importance of vision assessment and medication reviews. This study confirms that the program provides positive benefits and reduces fall risk factors among participants when implemented in multiple community-based settings in three states.

## Conflict of Interest Statement

The authors declare that the research was conducted in the absence of any commercial or financial relationships that could be construed as a potential conflict of interest.

This paper is included in the Research Topic, “Evidence-Based Programming for Older Adults.” This Research Topic received partial funding from multiple government and private organizations/agencies; however, the views, findings, and conclusions in these articles are those of the authors and do not necessarily represent the official position of these organizations/agencies. All papers published in the Research Topic received peer review from members of the Frontiers in Public Health (Public Health Education and Promotion section) panel of Review Editors. Because this Research Topic represents work closely associated with a nationwide evidence-based movement in the US, many of the authors and/or Review Editors may have worked together previously in some fashion. Review Editors were purposively selected based on their expertise with evaluation and/or evidence-based programming for older adults. Review Editors were independent of named authors on any given article published in this volume.

## References

[1] Stevens J Noonan R Rubenstein L Older adult fall prevention: perceptions, beliefs, and behaviors. Am J Lifestyle Med (2010) 4(1):16–20 10.1177/1559827609348350

[2] Tinetti M Baker D Garrett P Gottschalk M Koch M Horwitz R. Yale FICSIT: risk factor abatement strategy for fall prevention. J Am Geriatr Soc (1993) 41(3):315–20. 8440856 10.1111/j.1532-5415.1993.tb06710.x

[3] Rubenstein LZ. Falls in older people: epidemiology, risk factors and strategies for prevention. Age Aging (2006) 35(Suppl 2):ii37–41. 10.1093/ageing/afl084 16926202

[4] Stevens JA Baldwin GT Ballesteros MF Noonan RK Sleet DA. An older adult falls research agenda from a public health perspective. Clin Geriatr Med (2010) 26(4):767–79. 10.1016/j.cger.2010.06.006 20934621

[5] Clemson L Cumming RG Kendig H Swann M Heard R Taylor K. The effectiveness of a community-based program for reducing the incidence of falls in the elderly: a randomized trial. J Am Geriatr Soc (2004) 52(9):1487–94. 10.1111/j.1532-5415.2004.52411.x 15341550

[6] Wisconsin Institute for Healthy Aging. Stepping on an Implementation Guide: How to Prepare for, Implement, and Evaluate Stepping on in Community Settings. Centers for Disease Control and Prevention (2012). Available from: https://wihealthyaging.org/_data/files/SO_materials/Stepping-On-Manual_10-17-2013.pdf.

[7] Mahoney JE “Stepping On”: Stepping over the chasm from research to practice. Front Public Health (2015) 2:148 10.3389/fpubh.2014.00148 PMC4410405 25964898

[8] Stevens JA A CDC Compendium of Effective Fall Interventions: What Works for Community-Dwelling Older Adults. Atlanta, GA: Centers for Disease Control and Prevention. National Center for Injury Prevention and Control. Division of Unintentional Injury Prevention (2010).

[9] Kaniewski M Stevens JA Parker EM Lee R An introduction to the Centers for Disease Control and Prevention’s efforts to prevent older adult falls. Front Public Health (2015) 2:119 10.3389/fpubh.2014.00119 PMC4410411 25964892

[10] Wallace RB Herzog AR. Overview of the health measures in the health and retirement study. J Hum Resour (1995) 30:S84–107. 10.2307/14627920394273

[11] Hutton I Gamble G McLean G Butcher H Gow P Dalbeth N. Obstacles to action in arthritis: a community case-control study. Int J Rheum Dis (2009) 12(2):107–17. 10.1111/j.1756-185X.2009.01392.x 20374327

[12] Shumway-Cook A Brauer S Woollacott M Predicting the probability for falls in community-dwelling older adults using the timed up & go test. Phys Ther (2000) 80(9):896–903. 10960937

[13] Bohannon RW. Reference values for the timed up and go test: a descriptive meta-analysis. J Geriatr Phys Ther (2006) 29(2):64–8. 10.1519/00139143-200608000-00004 16914068

[14] Podsiadlo D Richardson S The timed “up & go”: a test of basic functional mobility for frail elderly persons. J Am Geriatr Soc (1991) 39(2):142–8. 1991946 10.1111/j.1532-5415.1991.tb01616.x

[15] Bischoff HA Stahelin HB Monsch AU Iversen MD Weyh A von Dechend M Identifying a cut-off point for normal mobility: a comparison of the timed ‘up and go’ test in community-dwelling and institutionalised elderly women. Age Ageing (2003) 32(3):315–20. 10.1093/ageing/32.3.315 12720619

[16] Tennstedt S Howland J Lachman M Peterson E Kasten L Jette A. A randomized, controlled trial of a group intervention to reduce fear of falling and associated activity restriction in older adults. J Gerontol B Psychol Sci Soc Sci (1998) 53(6):384–92. 10.1093/geronb/53B.6.P384 9826971

[17] Tinetti ME Baker DI McAvay G Claus EB Garrett P Gottschalk M A multifactorial intervention to reduce the risk of falling among elderly people living in the community. N Engl J Med (1994) 331(13):821–7. 10.1056/NEJM199409293311301 8078528

[18] Tinetti ME Richman D Powell L. Falls efficacy as a measure of fear of falling. J Gerontol (1990) 45(6):239–43. 10.1093/geronj/45.6.P239 2229948

[19] Vellas BJ Wayne SJ Romero LJ Baumgartner RN Garry PJ. Fear of falling and restriction of mobility in elderly fallers. Age Ageing (1997) 26(3):189–93. 10.1093/ageing/26.3.189 9223714

[20] Stevens JA Phelan EA Development of STEADI: a fall prevention resource for health care providers. Health Promot Pract [Internet]. (2012); [1–10 pp.]. Available from: http://www.ncbi.nlm.nih.gov/pubmed/23159993 10.1177/1524839912463576 PMC4707651 23159993

[21] National Center for Injury Prevention and Control. Tai chi: Moving for Better Balance a Guide for Program Implementation. (2011). p. 1–146 Available from: http://api.ning.com/files/lpWX79eu*NgqhrxYqum3lrPOm6Dp4GRQCK9J1qwtJylSBPaJ2W38NVNJNcBqszLkRplWB9ee-lwV4PIN-hUoPujtn*zU9vRw/TCManual_Compiled__v19.pdf

[22] Ory MG Smith ML Parker EM Jiang L Chen S Wilson AD Fall prevention in community settings: results from implementing Tai Chi: Moving for Better Balance in three states. Front Public Health (2015) 2:258. 10.3389/fpubh.2014.00258 25964934 PMC4410325

[23] American College of Sports Medicine. ACSM’s Guidelines for Exercise Testing and Prescription. 9 ed Baltimore, MD: Lippincott Williams & Wilkins (2013). 480 p. 10.1249/JSR.0b013e31829a68cf 23851406

[24] Centers for Disease Control and Prevention (CDC). STEADI (Stopping Elderly Accidents, Deaths & Injuries) Tool Kit for Health Care Providers Atlanta, GA, (2014). Available from: http://www.cdc.gov/homeandrecreationalsafety/Falls/steadi/index.html?s_cid=tw_injdir154.

